# Reference Genes for Expression Analyses by qRT-PCR in *Enterobacter cancerogenus*

**DOI:** 10.3390/microorganisms12051024

**Published:** 2024-05-19

**Authors:** Yang Pan, Yue Zhao, Hua-Rui Zeng, Jia-Qi Wu, Ying-Ying Song, Ya-Hao Rao, Guo-Qing Li, Lin Jin

**Affiliations:** Education Ministry Key Laboratory of Integrated Management of Crop Diseases and Pests/State & Local Joint Engineering Research Center of Green Pesticide Invention and Application, Department of Entomology, College of Plant Protection, Nanjing Agricultural University, Nanjing 210095, China; 2022202023@stu.njau.edu.cn (Y.P.); 2022102091@stu.njau.edu.cn (Y.Z.); 2021102074@stu.njau.edu.cn (H.-R.Z.); 2023102075@stu.njau.edu.cn (J.-Q.W.); 2019102080@stu.njau.edu.cn (Y.-Y.S.); 2023102074@stu.njau.edu.cn (Y.-H.R.); ligq@njau.edu.cn (G.-Q.L.)

**Keywords:** *Enterobacter cancerogenus*, qRT-PCR, reference genes, BestKeeper, NormFinder, geNorm, RefFinder

## Abstract

The *Enterobacter cancerogenus* strain EcHa1 was isolated from the dead larvae of *Helicoverpa armigera*, and has the potential for biocontrol of some Lepidoptera insects. In order to screen insecticidal-related genes by qRT-PCR, stable endogenous reference genes used for normalizing qRT-PCR data were selected and evaluated from 13 housekeeping genes (HKGs). The expression levels of the HKGs were determined using qRT-PCR under different experimental conditions, including two culture temperatures and three bacterial OD values. Five stability analysis methods (C_t_, BestKeeper, NormFinder, geNorm, and RefFinder) were used to comprehensively rank the candidate genes. The results showed that the optimal reference genes varied under different experimental conditions. The combination of *gyrA* and *gyrB* was recommended as the best reference gene combination at 28 °C, while *gyrA* and *rpoB* was the best combination at 37 °C. When the OD values were 0.5, 1.0 and 2.0, the recommended reference gene combinations were *ftsZ* and *gyrA*, *rpoB* and *gyrB*, and *gyrA* and *pyk*, respectively. The most suitable reference genes were *gyrA* and *gyrB* under all experimental conditions. Using *gyrA* and *gyrB* as the reference genes for qRT-PCR, EcHa1 was found to invade all tissues of the *H. armigera* larvae, and expressed a candidate pathogenic factor *Hcp* at high levels in gut, Malpighian tubules, and epidermis tissues. This study not only establishes an accurate and reliable normalization for qRT-PCR in entomopathogenic bacteria but also lays a solid foundation for further study of functional genes in *E. cancerogenus*.

## 1. Introduction

*Enterobacter cancerogenus* is a Gram-negative anaerobe bacillus [1,2]. It is widely distributed in nature [3], and has been successfully isolated from insect *Diprion pini* (Hymenoptera, Diprionidae) [4] and *Lutzomyia evansi* (Diptera, Psychodidae) [5] and plant tomato (*Solanum lycopersicum*) [6] and rice (*Oryza sativa*) [7]. A few reports suggest that *E. cancerogenus* may be a potential pathogen infecting patient skin and soft tissues when a wound exists and cause human diseases such as sepsis [8,9].

Recently, an *E. cancerogenus* strain, EcHa1, was isolated in the laboratory from the dead larvae of cotton bollworm, *Helicoverpa armigera* (Lepidoptera: Noctuidae) [10], a significant agricultural pest of cotton worldwide [11]. This was the first isolated strain belonging to *E. cancerogenus* to have high insecticidal activity against insect larvae. Excitingly, some strains of *Enterobacter* showed insecticidal, acaricidal, nematocidal, fungicidal, and plant growth-promoting activities, indicating that *Enterobacter* bacteria have biological control potential in agriculture [4,12,13]. Moreover, some strains contain type VI secretion systems (T6SS), and can establish competition by secreting antimicrobial proteins in the periplasm of bacterial targets [14]. These bacteria have the potential to develop antibacterial technology.

Nowadays, quantitative real-time fluorescent polymerase chain reaction (qRT-PCR), a well-established method that allows for the simultaneous detection and quantification of multiple target genes and organisms in a single sample [15], also offers a powerful tool for microbial detection. qRT-PCR not only enables rapid and sensitive bacterial identification but also aids in the study of the function of known pathogenic microorganisms [16]. However, the qRT-PCR technique has not been fully established in the detection of *Enterobacter* spp. In addition, various experimental errors during mRNA extraction, reverse transcription, and PCR performance can affect the accuracy of qRT-PCR [17]. In order to avoid these influences, it is necessary to combine relatively stable reference genes for normalization [18].

To facilitate the rapid detection of specific genes in *E. cancerogenus* EcHa1 and other *Enterobacter* spp. by qRT-PCR, it is essential to screen for reference genes that exhibit stable expression under various treatment conditions. In the current paper, we aimed to identify the most stably expressed reference genes in *E. cancerogenus* EcHa1 cultured at two different temperatures and three optical density (OD) values. Thirteen bacterial housekeeping genes (HKGs) (*gyrB*, *gyrA*, *era*, *secA*, *dnaG*, *ftsZ*, *RPSD*, *16S rRNA*, *rpoB*, *proC*, *pyk*, *rho* and *rplD*) were selected based on previously documented studies [19,20,21]. Four widely used analytical tools, i.e., C_t_ [22], geNorm [23], NormFinder [24], and BestKeeper [25], were used to assess the stability of candidate reference genes. Additionally, RefFinder [26,27] was employed to rank the stability of all the 13 HKGs. Based on the results, we recommended the most stable combinations of the internal reference genes for different experimental conditions. Our results will help to accurately detect the expression levels of the target genes in *E. cancerogenus* EcHa1 for future research and establish a set of methods for searching internal reference genes in *Enterobacter* bacteria.

## 2. Materials and Methods

### 2.1. Bacterial Strain

The *E. cancerogenus* strain EcHa1 (BioSample ID: SAMN16176831, https://www.ncbi.nlm.nih.gov/biosample/SAMN16176831/, accessed on 5 September 2023) used in this study, which was collected from the infected and dead larvae of *H. armigera* in Nanjing, China in 2021, was isolated in a laboratory by Nanjing Agricultural University [10]. The strain was grown normally in nutrient-rich Luria–Bertani (LB) medium [28] at 28 °C.

### 2.2. Bacterial Growth and Collection of Samples

Under normal conditions, *E. cancerogenus* EcHa1 was inoculated at a ratio of bacterial solution:medium = 1:1000 and cultured overnight (28 °C, 220 r.p.m. shaking) in LB broth provided by the MDBio company. Glycerol bacteria were prepared by adding 15% glycerol to the bacterial solution, which can be stored in the refrigerator at −80 °C for a long time. Glycerol bacteria were removed and cultured regularly to ensure bacterial viability. In this study, the culture temperature and bacterial OD values were used as variables to collect bacterial cells under different treatment conditions. The strains were inoculated in 5 mL LB broth and cultured at 28 °C and 37 °C until the bacterial OD values reached 0.5 after 1–2 h, 1.0 after 3–4 h, and 2.0 after 8–9 h. The bacterial cells were collected by centrifugation under six different treatment conditions (13,000× *g*, 15 min, 4 °C), and the supernatants were removed. The precipitated bacterial cells were used as an independent sample for RNA extraction from the cells. Each treatment was repeated three times to ensure confidence in the results.

### 2.3. Selection of Reference Genes

The sequences of thirteen HKGs (DNA gyrase subunit B, *gyrB*; DNA gyrase subunit A, *gyrA*; GTP-binding protein Era, *era*; protein translocase subunit SecA, *secA*; DNA primase, *dnaG*; cell-division protein FtsZ, *ftsZ*; RNA polymerase II subunit D, *RPSD*; 16S ribosomal RNA, *16s rRNA*; RNA polymerase beta subunit, *rpoB*; pyrroline-5-carboxylate reductase, *proC*; pyruvate kinase, *pyk*; Rho termination factor, *rho*; ribosomal protein L4, *rplD*) were selected. The sizes of the HKG sequences are listed in Table 1.

The identification of HKGs was performed using reverse-transcription polymerase chain reaction (RT-PCR), with the primers listed in Table 1. The primers were designed by the Primer3 website (https://bioinfo.ut.ee/primer3-0.4.0/, accessed on 15 October 2023), and the sequences of primers were submitted to Tsingke Biological Technology, Nanjing, China for synthesizing. To validate the specificity of the primer pairs, they were aligned against the entire genome sequence and compared with the sequencing results of individual amplified bands. Furthermore, to mitigate any potential impact from other intestinal bacteria in subsequent experiments, we cross-referenced the primer amplification sequences with all known species in NCBI (https://blast.ncbi.nlm.nih.gov/Blast.cgi, accessed on 15 October 2023), revealing only *E. cancerogenus* EcHa1 and its closely related counterparts. These findings affirmed that the primer pair exhibits excellent specificity and is suitable for future functional investigations of target genes (Table S1).

Total RNA of the bacterial cells was extracted using a Bacteria RNA Extraction Kit (Vazyme, Nanjing, China) and then reverse-transcribed using a HiScript^®^ II 1st Strand cDNA Synthesis Kit (+gDNA wiper) (Vazyme, Nanjing, China) according to the manufacturer’s protocols. For RT-PCR, the reverse-transcription process includes RNA template denaturation, genomic DNA removal, and first-strand cDNA synthesis. During the process, random hexamers were used as the reverse-transcription primer, and the reaction solution without RNA template was set as the negative control.

The PCR reaction mixture (final reaction volume of 25 μL) consisted of 9.5 μL of nuclease-free water, 12.5 μL of 2 × Rapid Taq Master Mix (Vazyme, China), 1 μL of forward primer (10 μM), 1 μL of reverse primer (10 μM), and 1 μL of cDNA template. The PCR protocol included an initial step of 95 °C for 30 s, followed by 35 cycles, each cycle including 95 °C for 30 s, 56 °C for 30 s, and 72 °C for 30 s, c, followed by one cycle of 72 °C for 8 min, and stored at 4 °C. The amplified products were separated by electrophoresis on a 1% agarose gel and purified utilizing the Wizard^®^ Preps PCR DNA Purification System (Promega, Madison, WI, USA). Following purification, the DNA was ligated into the pGEM^®^-T easy vector (Promega), and multiple independent subclones were sequenced bidirectionally. The obtained sequencing results were submitted to GenBank, and their corresponding accession numbers are shown in Table 1.

### 2.4. Quantitative Real-Time PCR (qRT-PCR)

The primers for qRT-PCR were designed using Beacon Designer 7 (Premier Biosoft International, Palo Alto, CA, USA), and the information of these primers is listed in Table S2. The sequences of these primers were also submitted to Tsingke Biological Technology, Nanjing, China for synthesizing. For qRT-PCR analysis, the reverse-transcription process includes genomic DNA removal and first-strand cDNA synthesis. ChamQ Universal SYBR qPCR Master Mix (Vazyme Biotech Co., Ltd.) was used to prepare the qRT-PCR reaction solutions according to the manufacturer’s protocol, and the QuantStudio™ 7 Pro Real-Time PCR System (Applied Biosystems, Thermo Fischer Scientific, Waltham, CA, USA) was used for performing the reactions. The reaction mixture (final reaction volume of 20 μL) consisted of 7.2 μL of nuclease-free water, 10 μL of 2 × ChamQ Universal SYBR qPCR Master Mix, 0.4 μL of forward primer (10 μM), 0.4 μL of reverse primer (10 μM), and 2 μL of cDNA template. Two negative controls were included for each primer set to confirm the absence of genomic DNA and to check for primer dimers or contamination in the reactions, one without reverse transcriptase and the other without template. The qRT-PCR protocol included an initial step of 95 °C for 30 s, followed by 40 cycles of 95 °C for 5 s and then annealing at 60 °C for 34 s, followed by one cycle of 95 °C for 15 s, 60 °C for 60 s, and 95 °C for 1 s. PCR amplicons were subjected for melting curve analysis. The specificity of the qRT-PCR reactions was monitored with melting curves, analyzed by QuantStudio™ Design & Analysis Software (version 1.5.0) and gel electrophoresis. Amplification efficiency was determined by a 10-fold dilution series of template. All experiments were repeated in triplicate.

### 2.5. Evaluation of Reference Gene Selection

The activated *E. cancerogenus* EcHa1 solutions were inoculated in LB medium to culture until the OD values reached 0.5, 1.0, and 2.0 (28 °C, 220 r.p.m. shaking). For bioassays, we used diet overlay bioassays to feed larvae of *H. armigera*. A liquid artificial diet (5 mL) was dispensed into each well of the six-well plate. After the diet cooled and solidified, 800 μL of each three bacteria with different OD values was applied evenly to the diet surface in each well and allowed to dry. A single fourth-instar larva with the same body size and growth was starved for 2 h and placed in each well to feed with *E. cancerogenus* for 24 h. Larvae were kept at 26 (±1) °C, 60% (±10%) relative humidity, and 16 h light: 8 h dark. A six-well plate was used as one replicate, with three replicates set for each treatment, and the control group was fed the LB medium. The tissue samples of the fat body, head capsule, gut, Malpighian tubules, epidermis and hemolymph of the larvae from different treatments were collected by dissection. The larvae were sterilized with 75% alcohol three times before dissection and washed with water after each sterilization. Total RNA of the tissues was extracted using a Bacteria RNA Extraction Kit (Vazyme, Nanjing, China) and then reverse-transcribed with a HiScript^®^ II 1st Strand cDNA Synthesis Kit (+gDNA wiper) (Vazyme, Nanjing, China) according to the manufacturer’s protocols. We used qRT-PCR to detect the raw C_t_ values of two reference genes in different tissues of larvae. The averages (±SE) of the raw C_t_ values were compared using Student’s *t* test between CK and treatment.

A candidate pathogenic factor, the structural gene *Hcp* (GenBank accession number: PP768336) encoding the inner tube protein of Type VI secretion system (T6SS) of EcHa1, was used to evaluate the stability of candidate reference genes. The sequence of the primers for qRT-PCR is listed in Table 1. We used qRT-PCR to detect the raw C_t_ values of two reference genes and *Hcp* in different tissues of larvae. The average relative levels of *Hcp* in different tissues of larvae were computed based on the 2^−∆∆Ct^ method and from five replicates. We used SPSS for Windows (Chicago, IL, USA) for statistical analyses. The averages (±SE) were submitted to analysis of variance with the Tukey–Kramer test.

### 2.6. Data Processing

QuantStudio™ Design & Analysis Software (version 1.5.0) was used to visualize the raw C_t_ values. In order to obtain the stability of the selected HKGs, three commonly used algorithms, geNorm [23], BestKeeper [25], and Normfinder [24] (https://blooge.cn/RefFinder/?type=reference, accessed on 31 October 2023), were used strictly to analyze the raw C_t_ values according to the manuals. The jvenn tool [29] was used to analyze the common stable genes from different conditions based on the results from each algorithm, respectively. The comprehensive ranking of HKGs at each condition was analyzed and evaluated according to the algorithm RefFinder [26,27]. In addition, the number of reference genes for normalizing gene expression was decided by the pairwise variation (Vn/n + 1), which was performed using the geNorm program. Universally, when Vn/n + 1 is less than the threshold value of 0.15, it indicates that the most suitable number of the reference genes is n, and there is no need to introduce the n + 1 reference gene for normalization [23].

## 3. Results

### 3.1. Selection of Candidate HKGs

Thirteen HKGs were selected and designated as *gyrB*, *gyrA*, *era*, *secA*, *dnaG*, *ftsZ*, *RPSD*, *16S rRNA*, *rpoB*, *proC*, *pyk*, *rho* and *rplD*. The resultant sequences were submitted to GenBank, and the accession numbers are listed in Table 1. The correctness of the 13 HKGs was proven by RT-PCR.

The products from RT-PCRs were confirmed by sequencing. Primer specificities for qRT-PCR were verified by melting curve analysis. All the primer pairs amplified a single PCR product with the expected sizes and sequences. The regression coefficients (R2) of the 13 HKGs ranged from 0.991 to 0.999, reaching the standard requirements of conventional qRT-PCR [30]. Moreover, the slopes were near −3.0 and efficacy values stretched from 88.74% to 141.42% (Table S2).

### 3.2. C_t_ Values of Candidate HKGs

The qRT-PCR revealed that all 13 HKGs were expressed during bacterial propagation at different OD values and temperatures, indicated by the presence of a single amplicon of the expected size on the agarose gel.

**Figure 1:**
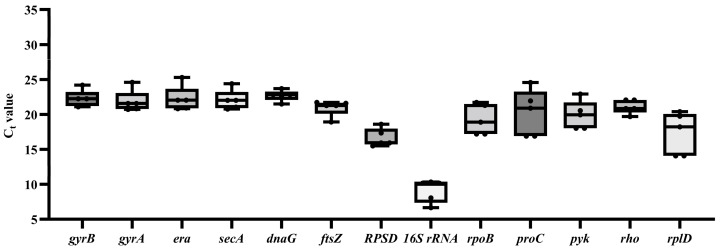
Expression levels of thirteen housekeeping genes in *E. cancerogenus*.

The overall threshold cycle (C_t_) values for all experimental conditions are shown in Figure 1. The boxplot results indicated that the expression levels of the 13 HKGs were variable. Under the given experimental conditions, the C_t_ values of the 13 HKG genes varied from 6.65 to 24.60, and the average C_t_ value ranged from 9.08 to 22.73. Among them, *16S rRNA* and *RPSD* had high expression levels, and the expression levels of other reference genes were similar (Figure 1).

### 3.3. BestKeeper Analysis

**Figure 2:**
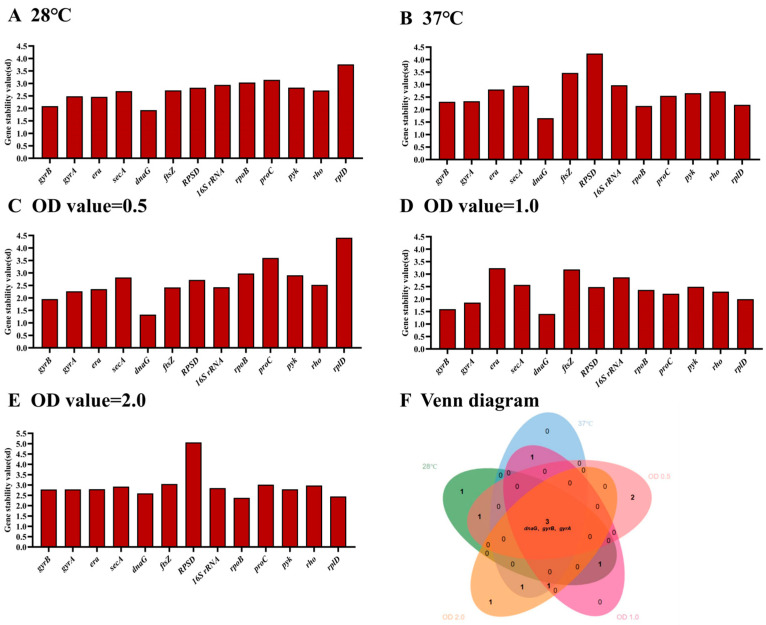
The stability of thirteen housekeeping genes in *E. cancerogenus* based on BestKeeper. The comparison was performed under two different experimental conditions including temperatures (**A**,**B**) and different OD values (**C**–**E**). The Venn diagram (**F**) shows the common stable genes from different conditions.

The online tool BestKeeper was used to rank the stability of the selected genes [25]. Using this analysis, the most stable HKGs were *dnaG*, *gyrB*, *era*, *gyrA* and *secA* at 28 °C (Figure 2A), and *dnaG*, *rpoB*, *rplD*, *gyrB* and *gyrA* at 37 °C (Figure 2B), respectively. In addition, the most stable HKGs were *dnaG*, *gyrB*, *gyrA*, *era* and *ftsz* at OD value of 0.5 of *E. cancerogenus* (Figure 2C), *dnaG*, *gyrB*, *gyrA*, *rplD* and *ProC* at OD value of 1.0 (Figure 2D), and *rpoB*, *rplD*, *dnaG*, *gyrB* and *gyrA* at OD value of 2.0 (Figure 2E), respectively. Obviously, *dnaG*, *gyrB* and *gyrA* were stable across different temperature and OD values (Figure 2F).

### 3.4. NormFinder Algorithm

The stability analysis of NormFinder [24] is based on the relative quantitative expression data of candidate reference genes, and a smaller stability value indicates greater gene expression stability.

**Figure 3:**
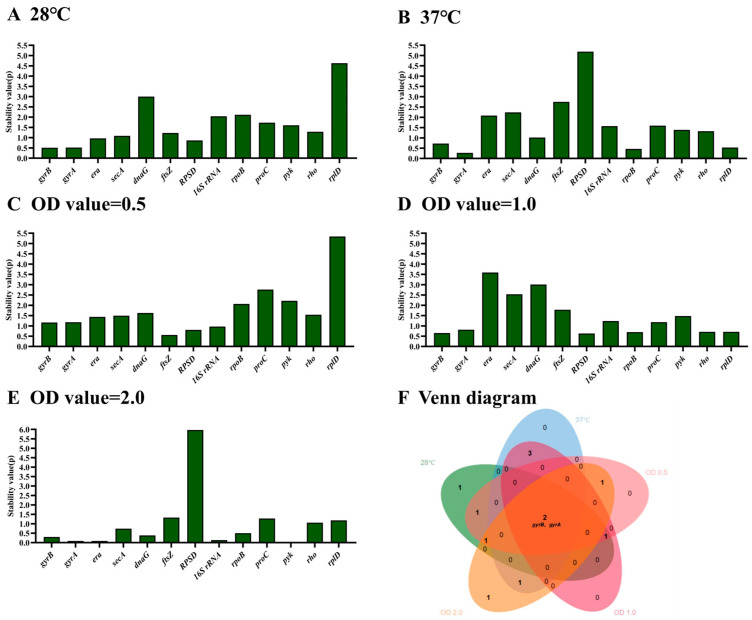
The stability of thirteen housekeeping genes in *E. cancerogenus* based on NormFinder. The comparison was performed under two different experimental conditions including temperatures (**A**,**B**) and different OD values (**C**–**E**). The Venn diagram (**F**) shows the common stable genes from different conditions.

According to NormFinder, the genes that are more stably expressed are indicated by lower average expression stability values. NormFinder analysis results revealed that the ranking of stability for the 13 HKGs from high to low was *rplD*, *dnaG*, *rpoB*, *16S rRNA*, *proC*, *pyk*, *rho*, *ftsZ*, *secA*, *era*, *RPSD*, *gyrA* and *gyrB* at 28 °C (Figure 3A), and *RPSD*, *ftsZ*, *secA*, *era*, *proC*, *16S rRNA*, *pyk*, *rho*, *dnaG*, *gyrB*, *rplD*, *rpoB* and *gyrA* at 37 °C (Figure 3B), respectively. The ranking of stability from high to low was *rplD*, *proC*, *pyk*, *rpoB*, *dnaG*, *rho*, *secA*, *era*, *gyrA*, *gyrB*, *16S rRNA*, *RPSD* and *ftsZ* when the OD value was 0.5 (Figure 3C), *era*, *dnaG*, *secA*, *ftsZ*, *pyk*, *16S rRNA*, *proC*, *gyrA*, *rho*, *rplD*, *rpoB*, *gyrB* and *RPSD* when the OD value was 1.0 (Figure 3D), and *RPSD*, *ftsZ*, *proC*, *rplD*, *rho*, *secA*, *rpoB*, *dnaG*, *gyrB*, *16S rRNA*, *gyrA*, *era* and *pyk* when the OD value was 2.0 (Figure 3E). The genes *gyrB* and *gyrA* were among the six most stable genes at different temperature and OD values (Figure 3F).

### 3.5. geNorm Method

Two parameters were defined by the geNorm statistical algorithm to quantify gene stability: M meaning the average expression stability and V meaning the pairwise variation. The HKG with the lowest M value can be considered to express most stably, while the one with the highest M value has the least stable expression.

**Figure 4:**
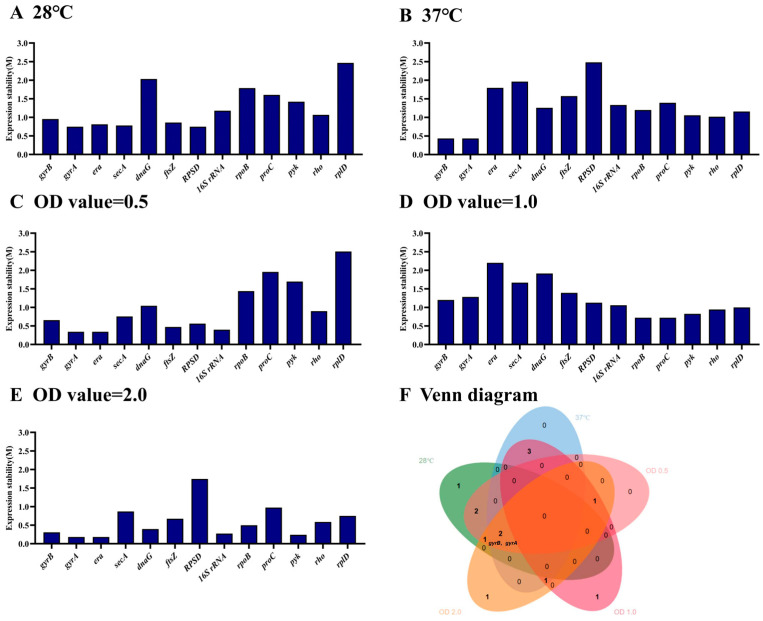
The stability of thirteen housekeeping genes in *E. cancerogenus* based on geNorm. The comparison was performed under two different experimental conditions including temperatures (**A**,**B**) and different OD values (**C**–**E**). The Venn diagram (**F**) shows the common stable genes from different conditions.

According to the M value, the most stable genes were *gyrA* and *RPSD* and the most unstable genes were *dnaG* and *rplD* at a culture temperature of 28 °C (Figure 4A). Comparably, the most stable genes were *gyrA* and *gyrB* (Figure 4B), respectively, at 37 °C. In addition, the most stable genes were *gyrA* and *era* (Figure 4C), *rpoB* and *proC* (Figure 4D) and *era* and *gyrA* (Figure 4E) at OD values of 0.5, 1.0, and 2.0 of *E. cancerogenus*, respectively. The most unstable genes were *secA* and *RPSD* (Figure 4B), *proC* and *rplD* (Figure 4C), *dnaG* and *era* (Figure 4D), and *proC* and *RPSD* (Figure 4E), respectively. The genes *gyrB* and *gyrA* were among the six most stable genes at different temperature and OD values (Figure 4F).

**Figure 5:**
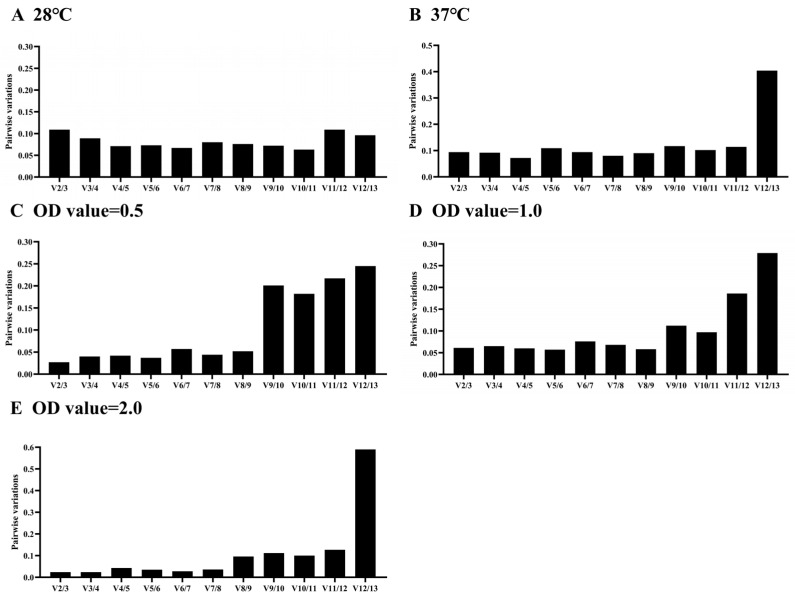
Optimal number of reference genes used for normalization of gene expression by geNorm. The comparison was performed under two different experimental conditions including temperatures (**A**,**B**) and different OD values (**C**–**E**).

A single reference gene may result in significant errors; therefore, the utilization of more than one reference gene is necessary. The Vn/Vn + 1 cutoff value serves as a crucial indicator for evaluating the optimal number of reference genes [23]. To ensure the utmost accuracy in qRT-PCR, multiple reference genes should be employed [31]. In this study, the V2/3 values were less than 0.15 at different temperature and OD values (Figure 5). This suggests that two reference genes were sufficient to analyze gene expression.

### 3.6. Best Combination of HKGs

**Figure 6:**
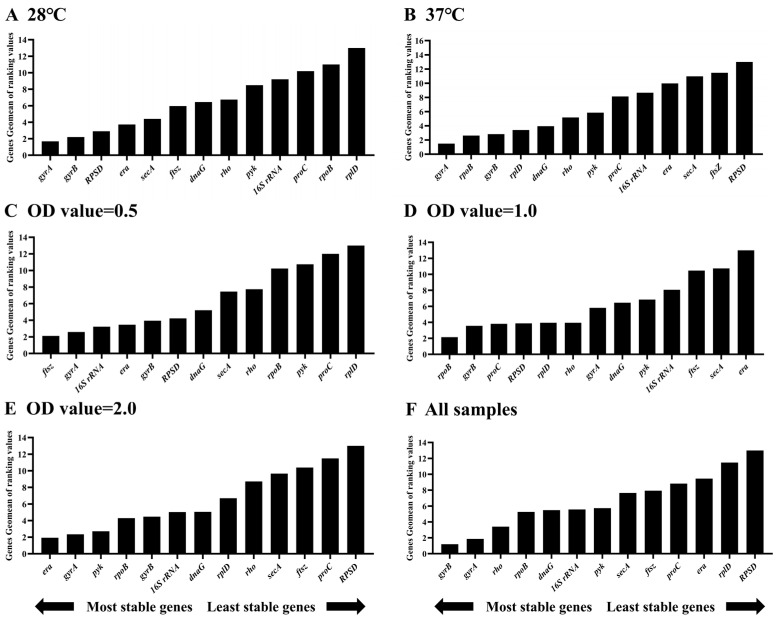
Expression stability of thirteen housekeeping genes in different samples of *E. cancerogenus*. The stability of the reference genes was calculated by the Geomean method of RefFinder. The comparison was performed under two different experimental conditions including temperatures (**A**,**B**), different OD values (**C**–**E**), and all samples (**F**).

In order to evaluate the stability of HKGs under different conditions and ensure statistical consistency and accuracy, a comprehensive ranking of the stability of these candidate genes was carried out using the RefFinder algorithm (Figure 6). RefFinder assigned different ranks to the analyzed HKGs under different conditions. Candidate genes with lower mean weights are considered stable and can be used as ideal reference genes [32].

The RefFinder analysis results revealed the ranking of stability for 13 HKGs from high to low was *gyrA*, *gyrB*, *RPSD*, *era*, *secA*, *ftsZ*, *dnaG*, *rho*, *pyk*, *16S rRNA*, *proC*, *rpoB* and *rplD* at 28 °C (Figure 6A), and *gyrA*, *rpoB*, *gyrB*, *rplD*, *dnaG*, *rho*, *pyk*, *proC*, *16S rRNA*, *era*, *secA*, *ftsZ* and *RPSD* at 37 °C (Figure 6B), respectively. The ranking of stability from high to low was *ftsZ*, *gyrA*, *16S rRNA*, *era*, *gyrB*, *RPSD*, *dnaG*, *secA*, *rho*, *rpoB*, *pyk*, *proC* and *rplD* when the OD value of *E. cancerogenus* was 0.5 (Figure 6C), *rpoB*, *gyrB*, *proC*, *RPSD*, *rplD*, *rho*, *gyrA*, *dnaG*, *pyk*, *16S rRNA*, *ftsZ*, *secA* and *era* when the OD value was 1.0 (Figure 6D), and *era*, *gyrA*, *pyk*, *rpoB*, *gyrB*, *16S rRNA*, *dnaG*, *rplD*, *rho*, *secA*, *ftsZ*, *proC* and *RPSD* when the OD value was 2.0 (Figure 6E). In all samples, the stability order was *gyrB*, *gyrA*, *rho*, *rpoB*, *dnaG*, *16S rRNA*, *pyk*, *secA*, *ftsZ*, *proC*, *era*, *rplD* and *RPSD* (Figure 6F). Given that two reference genes are sufficient to analyze gene expression, *gyrB* and *gyrA* were recommended as the reference genes during qRT-PCR in *E. cancerogenus* EcHa1.

### 3.7. Validation of the Selected Reference Genes

**Figure 7:**
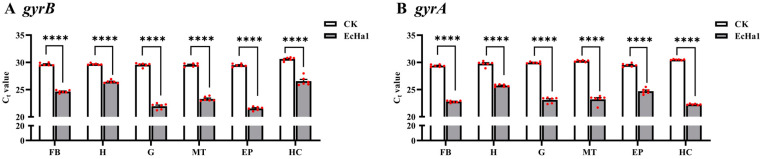
Expression levels of *gyrB* and *gyrA* genes in different tissues of larvae. The fat body (FB), head capsule (H), gut (G), Malpighian tubules (MT), epidermis (EP) and hemolymph (HC) were dissected from the larvae feeding with *E. cancerogenus* or LB medium for 24 h. For each sample, 5 independent pools of 20–30 individuals were measured in technical triplicate using qRT-PCR. The columns represent averages, with vertical lines indicating SE. The *t*-test was used to analyze the results, and the asterisks (****) indicate the significant difference (*p*-value < 0.01).

The threshold cycle (C_t_) values of *gyrB* and *gyrA* in the tissues of the fat body, head capsules, gut, Malpighian tubules, epidermis and hemolymph of *H. armigera* larvae were calculated by qRT-PCR. The results showed that after feeding the EcHa1 to the fourth-instar larvae for 24 h, the C_t_ values of *gyrB* and *gyrA* (Figure 7B) in different tissues of larvae were significantly lower than in tissues of larvae fed on LB medium. This indicates that EcHa1 infected all tissues of the larvae through the oral route.

**Figure 8:**
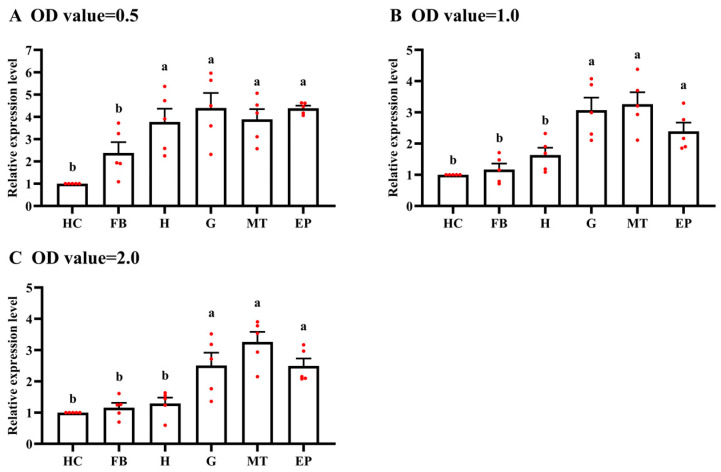
The relative expression of *Hcp* from *E. cancerogenus* in the different tissues of larvae. The fat body (FB), head capsule (H), gut (G), Malpighian tubules (MT), epidermis (EP) and hemolymph (HC) were dissected from the larvae feeding with *E. cancerogenus* of three different OD values. For each sample, 5 independent pools of 20–30 individuals were measured in technical triplicate using qRT-PCR. The values were calculated using the 2^−∆∆Ct^ method, using the selected reference genes *gyrB* and *gyrA*. The relative transcripts are the ratios of copy numbers in different treatments relative to the hemolymph, which is set as 1. The columns represent averages, with vertical lines indicating SE. Different letters indicate significant differences at *p* value < 0.01 using analysis of variance with the Tukey–Kramer test.

To demonstrate the utility of *gyrB* and *gyrA* in accurate gene expression analysis, the relative expression levels of a candidate pathogenic factor, *Hcp*, encoding the inner tube protein of EcHa1 T6SS in the fat body, head capsules, gut, Malpighian tubules, epidermis and hemolymph of larvae, were calculated after normalization with a combination of *gyrB* and *gyrA*. Compared with those in fat body and hemolymph, EcHa1 expressed *Hcp* at higher levels in gut, Malpighian tubules, and epidermis tissues of the fourth-instar larvae (Figure 8).

## 4. Discussion

The qRT-PCR technique has been extensively utilized in molecular biology due to its inherent advantages of precise, sensitive, and rapid quantification of gene expression [33]. It serves as the gold standard technique for detecting or comparing mRNA levels. To ensure accurate measurement of target gene mRNA level, it is generally imperative to calibrate using multiple validated reference genes [34]. In the present paper, we evaluated the stability of 13 candidate reference genes in *E. cancerogenus* EcHa1 under different temperatures and OD values using five widely employed analysis software tools (C_t_, BestKeeper, NormFinder, geNorm, and RefFinder).

In qRT-PCR, the C_t_ value is commonly utilized for assessing relative gene expression levels [35]. Based on the obtained C_t_ values in this study, it was observed that during the growth phase of *E. cancerogenus* EcHa1, the expression of *16S rRNA* exhibited a significant increase of more than three cycles (Figure 1). This finding aligns with previous research outcomes indicating high expression levels of *16S rRNA* across various experimental settings [20,36,37]. Except for *16S rRNA*, the expression levels of *RPSD* and *rplD* were also found to be higher compared to other candidate genes, while *era* exhibited the lowest expression level (Figure 1). Genes with extremely high or low transcript abundance are not suitable as standardization agents for genes with low or high expression levels, respectively [38]. Furthermore, all four tools (BestKeeper, NormFinder, geNorm, and RefFinder) consistently indicated that *RPSD*, *rplD*, and *era* were the least stable under different temperatures and OD values (Figure 2, Figure 4 and Figure 6). These findings suggest that these four genes should be excluded from consideration as reference genes for *E. cancerogenus* EcHa1. Similarly, rplD showed significant differences in the expression in *Listeria monocytogenes* under various stress adaptation models [39].

Relying on a single reference gene for normalization can result in inaccurate outcomes and more pronounced errors under specific experimental conditions [37]. Numerous studies have emphasized the significance of employing multiple stably expressed reference genes to achieve precise quantification of target gene expression [40,41,42], as an inadequate or excessive number of reference genes can compromise accuracy [43]. Therefore, it is essential to adjust the number of internal references based on the specific circumstances. In this study, geNorm analysis determined that two stably expressed reference genes were sufficient for analyzing gene expression in *E. cancerogenus* EcHa1 across different experimental conditions (Figure 5). Consequently, we needed to select two relatively stable reference genes from the remaining nine candidate genes (*gyrB*, *gyrA*, *secA*, *dnaG*, *ftsZ*, *rpoB*, *proC*, *pyk* and *rho*).

Using different tools for results analysis may yield varying outcomes, which can be attributed to the distinct algorithms employed by each tool. A similar scenario was observed in the evaluation of reference genes in *Bacillus cereus* [44]. In this study, variations were noted in the results obtained from three different tools’ analyses (BestKeeper, NormFinder and geNorm) (Figure 2, Figure 3 and Figure 4). Consequently, RefFinder was utilized to comprehensively rank each candidate reference gene (Figure 6). RefFinder analysis revealed that *gyrA* and *gyrB* constituted an ideal combination of reference genes. Consistently, *gyrB*, *gyrA*, *era*, *secA* and *dnaG* are commonly expressed with high stability across bacterial phyla [45]. Both *gyrA* and *gyrB* encode DNA gyrase enzymes that are capable of regulating the topological conformation of DNA molecules. GyrA is responsible for DNA cleavage and ligation, and GyrB contains ATP-binding sites [46]. DNA gyrase subunits have been recommended as reference genes in several bacterium species, including *Oenococcus oeni* [47], *Shewanella psychrophila* [36], *Dwardsiella tarda* [48], *Xanthomonas fragariae* [42], *Bacillus velezensis* [49], *Corynebacterium pseudotuberculosis* [19] and *Herbaspirillum rubrisubalbicans* [50].

The determination of the reference genes will be helpful to accurately detect the tissue distribution of entomopathogenic bacteria in insect hosts and the expression level of candidate pathogenic factors. This is of great significance for understanding the insecticidal mechanism of entomopathogenic bacteria and evaluating whether they have the potential for developing microbial insecticides. Entomopathogenic bacteria and/or their toxins must be ingested and enter the alimentary tract of insects where they multiply or are activated to initiate disease. Released bacterial toxins and other virulence factors target the midgut cells to disrupt the epithelial barrier and break through to the main body cavity [51]. Using the reference genes *gyrA* and *gyrB* screened in this study, we determined the distribution of *E. cancerogenus* EcHa1 in different tissues of *H. armigera* larvae after feeding the bacteria. The results showed that EcHa1 invaded all tissues of the larvae (Figure 7), indicating that EcHa1 was able to overcome a series of defensive mechanisms controlled by larvae and break through to the main body cavity to kill hosts. In addition, we used *gyrA* and *gyrB* to detect the relative expression of a candidate pathogenic factor, the structural gene *Hcp* encoding the inner tube protein of Type VI secretion system (T6SS) of EcHa1, in different tissues of larvae. Compared with those in fat body and hemolymph, EcHa1 expressed *Hcp* at a high level in gut, Malpighian tubules, and epidermis tissues of the fourth-instar larvae (Figure 8), suggesting that EcHa1 may destroy intestinal cells, Malpighian tubule cells and epidermal cells through T6SS, while cells in hemolymph and fat body may be destroyed by other ways, because these cells have strong innate immunity. It was reported based on reference genes for PCR normalization that the relative expressions of some genes associated with virulence and pathogenicity of *Pseudomonas protegens*, an entomopathogenic bacterium against muscoid flies by oral infection, were found to significant increase at the gut level of larvae after exposure to the bacterium [52]. Therefore, the relative expression of virulence factors detected by reference genes can be used to analyze the pathogenic mechanisms of pathogenic bacteria and evaluate the virulence level against pests.

What is limited in this study is that the stable reference gene combination *gyrA* and *gyrB* is only screened in a single Gram-negative bacterial strain EcHa1 under different temperatures and different bacterial contents. Whether this combination is suitable for other conditions, such as different environmental conditions or interaction between *E. cancerogenus* and target insect species, as a reference gene to normalize virulence gene expression level has not been clarified. Nevertheless, we used the reference genes to detect the expression level of candidate pathogenic factor *Hcp* after larvae had fed on EcHa1, and found that the difference in expression level was consistent with our prediction of the EcHa1 infection path, suggesting that the expression level of the reference genes was stable under different conditions. However, we suggest that when evaluating the relative expression level of functional genes of *E. cancerogenus*, these two reference genes should be used for standardization or combined with the results of protein quantitative analysis to render the conclusions accurate. In addition, the reference genes can only be used to judge whether there are *E. cancerogenus* in different tissues of insects, but it is impossible to evaluate the relative number because the structure of insect tissues is different. Therefore, in order to accurately analyze the function of bacterial pathogenic factors in the interaction with target insects, the structure and cell numbers of insect tissues should also be taken into account.

## 5. Conclusions

A set of stable housekeeping genes that can serve as suitable reference genes for *E. cancerogenus* have successfully been identified by four different analysis tools (BestKeeper, NormFinder, geNorm, and RefFinder). To the best of our knowledge, this study represents the first comprehensive evaluation of the reference genes in *E. cancerogenus*. The findings provide a solid foundation for future molecular investigations in *E. cancerogenus*, and provide a method for searching internal reference genes in *Enterobacter* bacteria.

## Figures and Tables

**Table 1:** The list of primers used for RT-PCR of the genes.

Gene Name	Primer Sequences (5′ to 3′)	Sequence Size of Complete Gene (bp)	Accession Number
*gyrB*	F-ATGTCGAATTCTTATGACTCCTCC R-TTAGATATCAATATTCGCCGCTT	2412	OR922791
*gyrA*	F-ATGAGCGACCTTGCGAGA R-TTACTCGTCTGCGTCATC	2637	OR922792
*era*	F-ATGAGCGAAGAAAAAACC R-CTACTGGTCTTCGCCGTA	906	OR922793
*secA*	F-ATGCTAATCAAATTATTAACCAAAG R-TTAGCTCAGGCGGCCGTG	2706	OR922794
*dnaG*	F-ATGGCTGGACGAATCCCA R-TCATTTTTTTTCAAGGGC	1743	OR922795
*ftsZ*	F-ATGTTTGAACCTATGGAACTGACC R-TTAGTCAGCTTGCTTACGCAGG	1152	OR922796
*RPSD*	F-ATGGCAAGATATTTGGGTCC R-TTACTTGGAGTAAAGCTCGA	621	OR922797
*16S rRNA*	F-ATTGAACGCTGGCGGCAGGCCTAA R-GCAGGTTCCCCTACGGTTACCTTG	1493	OR922798
*rpoB*	F-ATGGTTTACTCCTATACCG R-TTACTCGTCTTCCAGTTCG	4029	OR922799
*proC*	F-ATGGATAAGAAAATCGGGT R-TCAGGATTTACTGAGCGCC	810	OR922800
*pyk*	F-ATGTCCAGAAGGCTTCGCA R-TTACTCGACCGTCATAACG	1443	OR922801
*rho*	F-ATGAATCTTACCGAATTAAAG R-TTACGAGCGTTTCATCATATCG	1260	OR922802
*rplD*	F-ATGGAATTAGTATTGAAAGACGCGC R-TCATGCCAGCATCTCCTCAACT	606	OR922803
*Hcp*	F-ATGGCTGATACGTTCCAGAATG R-TTATTTCGGGGCAAGCGC	492	PP768336

## Data Availability

Data generated in association with this study are available in the Supplementary Materials online with this article.

## Supplementary Materials

The following supporting information can be downloaded at https://www.mdpi.com/article/10.3390/microorganisms12051024/s1. Table S1: Specificity verification of 13 candidate housekeeping genes; Table S2: Primers of 13 candidate housekeeping genes and pathogenic factor Hcp used in qRT-PCR.
