# Supplementary material for: Reference Genes for Expression Analyses by qRT-PCR in Enterobacter cancerogenus
Source: Microorganisms. 2024 May 19;12(5):1024. doi: 10.3390/microorganisms12051024 (PMC11123693; doi:10.3390/microorganisms12051024)
Supplement: Supplementary file 1 [file microorganisms-12-01024-s001.zip › microorganisms-2956645-supplementary.pdf]

Supplementary Materials

Reference genes for expression analyses by qRT-PCR in *Enterobacter cancerogenus*

Yang Pan, Yue Zhao, Hua-Rui Zeng, Jia-Qi Wu, Ying-Ying Song, Ya-Hao Rao, Guo-Qing Li and Lin Jin *

Table S1. Specificity verification of 13 candidate house-keeping genes.

Gene name	Description	Per. Ident	Accession number
gyrB	*E. cancerogenus* EcHa1	100.00%	CP082280.1
gyrA	*E. cancerogenus* 3.2.17	100.00%	CP134403.1
	E. cancerogenus EcHa1		CP082280.1
era	*E. cancerogenus* 3.2.17	100.00%	CP134403.1
	E. cancerogenus EcHa1		CP082280.1
secA	*E. cancerogenus* 3.2.17	100.00%	CP134403.1
	E. cancerogenus EcHa1		CP082280.1
dnaG	*E. cancerogenus* EcHa1	100.00%	CP082280.1
ftsZ	*E. cancerogenus* 3.2.17	100.00%	CP134403.1
	E. cancerogenus EcHa1		CP082280.1
RPSD	*E. cancerogenus* 3.2.17	100.00%	CP134403.1
	E. cancerogenus EcHa1		CP082280.1
16S rRNA	*E. cancerogenus* EcHa1	100.00%	CP082280.1
rpoB	*E. cancerogenus* 3.2.17	100.00%	CP134403.1
	E. cancerogenus EcHa1		CP082280.1
proC	*E. cancerogenus* EcHa1	100.00%	CP082280.1
pyk	*E. cancerogenus* EcHa1	100.00%	CP082280.1
rho	*E. cancerogenus* EcHa1	100.00%	CP082280.1
rplD	*E. cancerogenus* MiY-F	100.00%	CP045769.1
	E. cancerogenus CR-Eb1		CP025225.1
	E. cancerogenus 3.2.17		CP134403.1
	E. cancerogenus EcHa1		CP082280.1
	E. cancerogenus JY65		CP081105.1
	E. cancerogenus FDAARGOS 1428		CP077290.1
	E. cancerogenus UPC1		LR881936.1

Table S2. Primers of 13 candidate house-keeping genes and pathogenic factor Hcp used in qRT-PCR.

Gene	Primer sequences (5' to 3')	Length of amplicon sequence (bp)	Slope	R^2	Efficiency (%)
gyrB	F- CTGAAAGCGCTGGTGTATCA<br>R- GTGCTGTTCGTTCTCGTTCA	114	-3.2358	0.992	103.72
gyrA	F- GGTAAATATCACCCGCATGG<br>R- CAGCATGTAACGCAAGGAGA	84	-3.1297	0.995	108.70
era	F- GGCAAATCCACCCTGTTAAA<br>R- TTCAGTATGGATGCCCACAA	102	-3.3982	0.996	96.91
secA	F- GCGTACCGGTGAAGGTAAAA<br>R- TCACGCTGTGCCAGATAGTC	111	-2.6125	0.937	141.42
dnaG	F- TACCGGGCAGCTTTTAGAGA<br>R- TCGTTCCAGGCTGAGAGTTT	75	-2.9280	0.998	119.55
ftsZ	F- GCAAGAAGCGTATGGCTTTC<br>R- AAGCGTCAAGCAGGGAGATA	129	-3.1010	0.999	110.12
RPSD	F- AGGGCACCGACTTATTCCTT<br>R- TTTTGCTTTTCACGCAACTG	136	-3.6251	0.994	88.74
16S rRNA	F- CGATCCCTAGCTGGTCTGAG<br>R- GTGCAATATTCCCCACTGCT	93	-3.1003	0.997	110.16
rpoB	F- GATCAACTCCCTGTCCGTGT<br>R- GAGTTCGCCTGAGCGATAAC	150	-2.7870	0.992	128.46
proC	F- GCTGGTCAATGCTGGTATGA<br>R- CACATCGGCAATGTCTTCAG	70	-2.8402	0.991	124.95
pyk	F- TCAGTTCGACAACGTGGAAG<br>R- ATACGGGAGGTCATCAGTGC	126	-3.3120	0.991	100.42
rho	F- ACTGCGTACTGATGGTGCTG<br>R- GATGCTGGTTCGTCAAAGGT	109	-3.2561	0.997	102.82
rplD	F- ATGGCTCTGGAAGATGTGCT<br>R- CGTACGTCAACCTTGTGCAG	89	-2.7904	0.993	128.23
Hcp	F-TCTTCTGAACCGTCTCCTGC<br>R-AACTGCCCCTTAAGCTCCTT	177	-3.1726	0.999	106.63
